# Association Between the Access Site for Coronary Angiography and Catheter-induced Coronary Artery Dissection

**DOI:** 10.1016/j.jscai.2023.100606

**Published:** 2023-03-04

**Authors:** Pietro Di Santo, Paul W. Boland, Omar Abdel-Razek, Trevor Simard, Richard G. Jung, Simon Parlow, Pouya Motazedian, Joanne Joseph, Pascal Theriault-Lauzier, Ali Alomar, Ali Hillani, Saad Alhassani, Mohamed Bayoumi Ali, Kwadwo Kyeremanteng, Doug Coyle, Dean Fergusson, George A. Wells, Michael Froeschl, Marino Labinaz, Juan J. Russo, Benjamin Hibbert

**Affiliations:** a CAPITAL Research Group, Division of Cardiology, University of Ottawa Heart Institute, Ottawa, Ontario, Canada; b Faculty of Medicine, University of Ottawa, Ottawa, Ontario, Canada; c School of Epidemiology and Public Health, University of Ottawa, Ottawa, Ontario, Canada; d Division of Cardiology, Faculty of Medicine, Memorial University, St. John’s, Newfoundland and Labrador, Canada; e Department of Cardiovascular Diseases, Mayo Clinic School of Medicine, Rochester, Minnesota; f Department of Cellular and Molecular Medicine, University of Ottawa, Ottawa, Ontario, Canada; g Department of Critical Care Medicine, The Ottawa Hospital, Ottawa, Ontario, Canada; h Ottawa Hospital Research Institute, Ottawa, Ontario, Canada; i Cardiovascular Research Methods Centre, University of Ottawa Heart Institute, Ottawa, Ontario, Canada

**Keywords:** catheter-induced coronary artery dissection, coronary angiography, percutaneous coronary intervention, transfemoral approach, transradial approach

## Abstract

**Background:**

Catheter-induced coronary artery dissection (CICAD) is a rare complication of coronary angiography. The association between access site and CICAD remains unclear; however, transradial access (TRA) may be associated with a higher incidence of CICAD due to access vessel tortuosity and the mechanical disadvantage of catheters designed for the transfemoral access (TFA) approach.

**Methods:**

In this retrospective study, the reports of consecutive left heart catheterizations between April 2007, and December 2021 were reviewed for CICAD. Patients were excluded if the procedural report did not report an arterial access site. Identified CICAD cases were reviewed in detail.

**Results:**

There were 142/89,876 (0.16%) identified cases of CICAD. The access site was not associated with an increased risk of CICAD (0.18% with TRA vs 0.15% with TFA; relative risk [RR], 1.18; 95% CI, 0.84-1.65; *P* = .34) over the entire study period. With respect to TRA-related CICAD, male sex was associated with a decreased risk of dissection (RR, 0.64; 95% CI, 0.41-0.99; *P* = .04), but ST-elevation myocardial infarction at presentation was associated with an increased risk (RR, 3.01; 95% CI, 1.86-4.85; *P* < .01). In the TFA-predominant era, TRA was associated with an increased risk of CICAD (0.48% TRA vs 0.11% TFA; RR, 3.42; 95% CI, 2.05-5.69; *P* < .01)—an association that was not present in the TRA-predominant era. In-hospital mortality in patients with CICAD was 8.5%.

**Conclusions:**

CICAD is a rare complication of coronary angiography. Over a 15-year period, we did not demonstrate an association between access site and an increased risk of CICAD. There is substantial mortality associated with CICAD.

## Highlights

• Catheter-induced coronary artery dissection is a feared complication of coronary angiography.
• The access site was not associated with increased risk of catheter-induced coronary artery dissection.
• Bailout stenting of the affected coronary artery is the most common treatment for catheter-induced coronary artery dissection.
• Catheter-induced coronary artery dissection is associated with in-hospital mortality of 8.5%.

## Central Illustration

**Figure:**
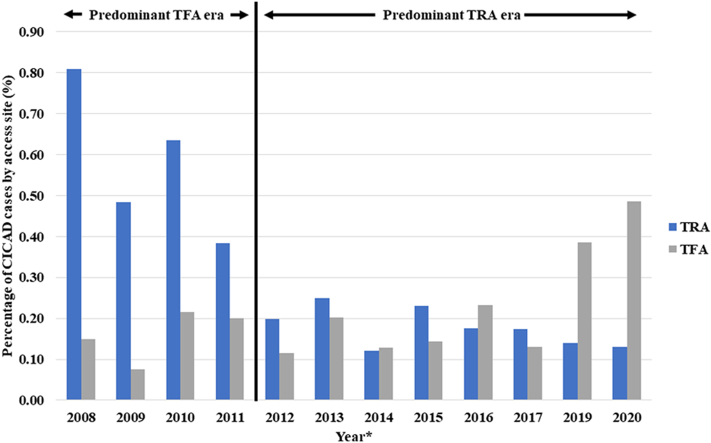

## Introduction

Transradial access (TRA) has emerged over transfemoral access (TFA) as the preferred approach for coronary angiography. The advantages of TRA include lower access site complications, earlier patient ambulation, and improved patient comfort.^1^ Several randomized control trials and meta-analyses comparing TRA to TFA in acute coronary syndrome have shown lower bleeding complications and reduced mortality favoring TRA.^2^, ^3^, ^4^, ^5^, ^6^

Although there are numerous advantages to TRA, there are disadvantages associated with this access site that must be considered. Patient factors include difficulty obtaining radial artery access, radial artery spasm, and difficulty with navigating tortuous upper extremity arterial anatomy. The catheters used for coronary angiography and percutaneous coronary angiography (PCI) were designed for use with TFA, contributing to increased difficulty with catheter manipulation and coronary intubation when utilized from a radial approach. Initial concerns about increased procedural failure, contrast use, and radiation exposure have been mitigated by studies showing that these parameters improve with operator experience.^7^^,^^8^

Catheter-induced coronary artery dissection (CICAD) is a rare complication of coronary angiography and PCI. The incidence of left main involvement was estimated between 0.03% and 0.07% in a large case series,^9^, ^10^, ^11^ whereas the incidence of right coronary artery CICAD is estimated at 0.14%.^12^ In a large contemporary series, the incidence of CICAD was 0.09% and most common with guide catheters.^13^ Anecdotally, TRA is accompanied by an increased risk of CICAD, theoretically due to access vessel tortuosity and the mechanical disadvantage of catheters designed for TFA.

In the retrospective study herein, we aim to compare the risk of CICAD in TRA versus TFA, as well as describe the risk factors, management, and clinical outcomes of CICAD at a large academic cardiac care center.

## Methods

Reports of consecutive cardiac catheterization procedures at a large quaternary care cardiac center between April 2007 and December 2021 were obtained. Patients could be included more than once if they had repeated catheterizations performed during the study period. Patients were excluded if the procedural report did not report an arterial access site. Reports with identified dissection were reviewed for the mechanism of dissection (eg, catheter-induced, spontaneous coronary artery dissection, postintervention stent edge dissection, etc.). Procedures involving transbrachial access were treated as TRA for the purpose of analysis. The study complied with the Declaration of Helsinki and was approved by the Ottawa Health Sciences Network Research Ethics Board. This research did not receive any specific grant from funding agencies in the public, commercial, or not-for-profit sectors.

A “catheter-induced coronary artery dissection” was defined as an unplanned dissection of the left main, right coronary artery, left internal thoracic artery, right internal thoracic artery, or aortic dissection/hematoma for which the catheter was identified as the cause by the operator’s procedural report. In cases where both TRA and TFA were reported as being accessed, CICAD cases were further reviewed to identify the arterial access resulting in the dissection. Procedural details, including equipment information such as catheter type, size, and shape, were obtained from the procedure log. Other key data, including age, height, weight, sex, procedure date, cardiovascular risk factors, operators, and presence of ST-elevation myocardial infarction were also collected from the catheterization reports and chart review.

All CICAD cases were further reviewed by 2 interventional cardiologists (P.D.S. and O.A.R.), and the coronary artery dissections were classified as types A to F based on the National Heart, Lung, and Blood Institute (NHLBI) system developed from the Coronary Angioplasty Registry.^14^ The type of dissection was based on the angiographic appearances of the intimal disruption and contrast clearance:

1. Type A dissections represent radiolucent areas within the coronary lumen during contrast injection, with minimal or no persistence of contrast.
2. Type B dissections are parallel tracts or double lumen separated by a radiolucent area during contrast injection, with minimal or no persistence.
3. Type C dissections appear as contrast outside the coronary lumen with persistence of contrast in the area after clearance of contrast from the coronary lumen.
4. Type D dissections represent spiral luminal filling defects, frequently with extensive contrast staining of the vessel.
5. Type E dissections appear as new, persistent filling defects.
6. Type F dissections represent those that lead to total occlusion of the coronary artery, without anterograde flow.

In cases where the angiographic images were not available for review, the detailed angiographic report was reviewed by 2 interventional cardiologists (P.D.S. and P.B.) to adjudicate if the dissection was felt to represent a catheter-induced dissection rather than by guide wires, intravascular imaging catheters, and/or postintervention complications (eg, postballoon dissection, distal stent edge dissection, etc.).

The primary outcome of this study was the incidence of CICAD by TRA vs TFA access sites. Secondary outcomes included evaluating risk factors for CICAD by access site. We also describe the management and clinical outcomes of patients with CICAD.

Outcomes were assessed using unadjusted χ^2^ analyses to compare the CICAD and non-CICAD groups, and corresponding relative risks (RRs) were generated along with 95% CIs. A sensitivity analysis was performed to evaluate the association between CICAD and access site stratified according to the most frequent access site utilized at our institution in a given year—a predominant TFA era (ie, prior to 2012) and a predominant TRA era (ie, 2012-2021; “radial first”). All statistical analyses were performed with the use of SAS software, version 9.4 (SAS Institute).

## Results

**Figure 1:**
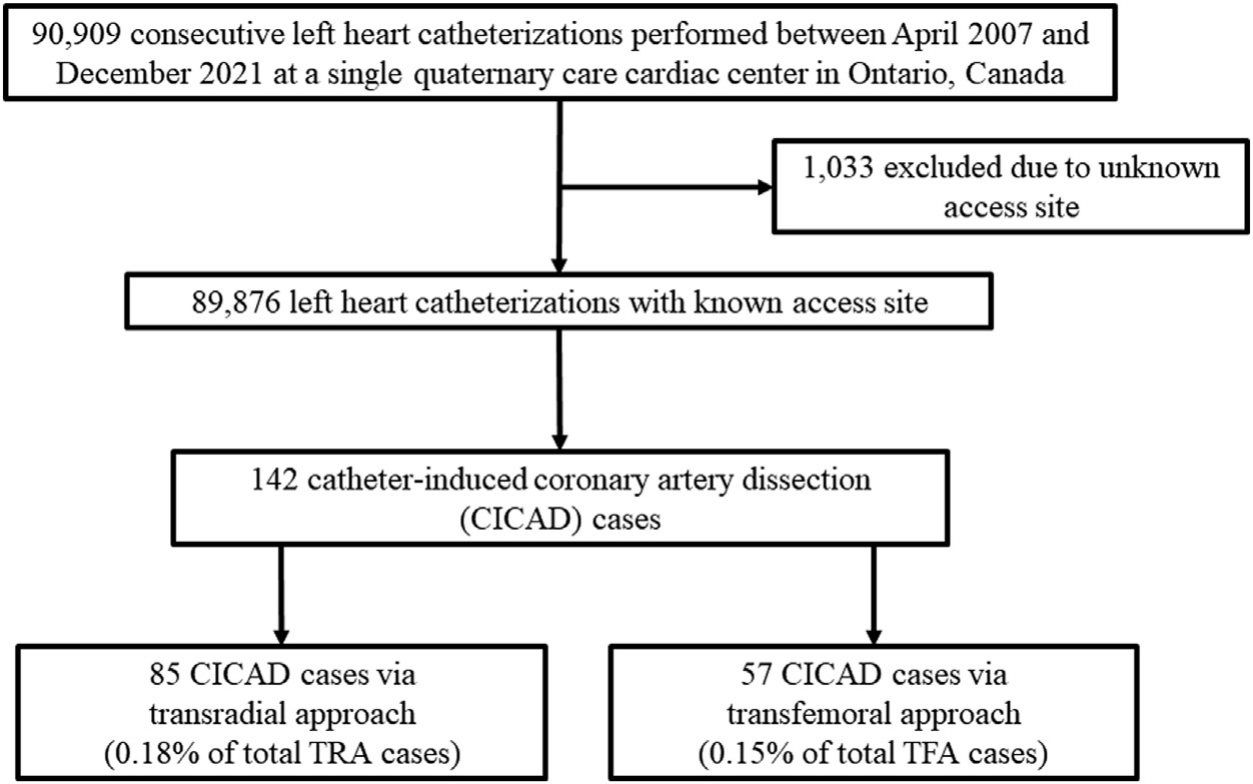
**Flow diagram of study patients**. CICAD, catheter-induced coronary artery dissection; TFA, transfemoral access; TRA, transradial access.

A total of 90,909 consecutive cardiac catheterizations were performed between April 2007 and December 2021. There were 1033 catheterizations without information on arterial access, which were excluded (Figure 1). The baseline characteristics of the entire study cohort as well as cases with and without CICAD are presented in Table 1. The mean age of the study patients was 66.1 ± 12.1 years, and most patients were males (68.2% across the entire study). Traditional cardiovascular risk factors were similar between the CICAD and non-CICAD groups.

**Table 1:** Baseline demographic characteristics of overall cohort as well as patients with and without catheter-induced coronary artery dissection.

	Left heart catheterization (N = 89,876)	CICAD (n = 142)	Non-CICAD (n = 89,734)
Age, y	66.1 ± 12.1	65.5 ± 12.9	65.9 ± 12.1
Male sex	60,956 (68.2%)	89 (63.1%)	60,867 (68.2%)
Height, m	1.70 ± 0.10	1.69 ± 0.11	1.70 ± 0.10
Weight, kg	83.8 ± 19.8	81.8 ± 17.9	83.4 ± 19.8
Medical history			
Hypertension	30,005 (33.4%)	43 (30.3%)	29,962 (33.4%)
Dyslipidemia	25,440 (28.3%)	30 (21.1%)	25,410 (28.3%)
Diabetes	23,047 (25.6%)	46 (32.4%)	23,001 (25.6%)
Active smoking	15,556 (17.3%)	24 (16.9%)	15,532 (17.3%)
Family history of premature CAD	4428 (4.9%)	3 (2.1%)	4425 (4.9%)
Procedural details			
STEMI at presentation	10,071 (11.2%)	32 (22.5%)	10,039 (11.2%)
Transradial access	47,826 (53.2%)	85 (59.9%)	47,741 (53.2%)
Transfemoral access	37,778 (42.0%)	57 (40.1%)	37,721 (42.0%)
Multiple access sites during procedure	4272 (4.8%)	30 (21.1%)	4272 (4.8%)
PCI during procedure	38,985 (43.4%)	115 (81.0%)	38,870 (43.3%)

Values are mean ± SD or n (%).

CAD, coronary artery disease; CICAD, catheter-induced coronary artery dissection; PCI, percutaneous coronary intervention; STEMI, ST-elevation myocardial infarction.

A total of 142 cases were found to have CICAD (Table 2). The access site was not associated with an increased risk of CICAD (0.18% of all TRA cases vs 0.15% of all TFA cases; RR, 1.18; 95% CI, 0.84-1.65; *P* = .34). Of note, there was no difference in CICAD between right and left radial artery TRA procedures (*P* = .52). NHLBI classification was available for 54 cases of CICAD with type B and F dissections representing two-thirds of dissection cases (Supplemental Table 1). Age was not associated with TRA-related CICAD (*P* = .86) or TFA-related CICAD (*P* = .30). With respect to TRA-related CICAD, male sex was associated with a decreased risk of dissection (RR, 0.64; 95% CI, 0.41-0.99; *P* = .04), but not with TFA (*P* = .84). ST-elevation myocardial infarction was associated with an increased risk of dissection in TRA (RR, 3.01; 95% CI, 1.86-4.85; *P* < .01) but not with TFA (RR, 1.46; 95% CI, 0.72-2.97; *P* = .30). The right coronary artery was the most affected artery in both the TRA-related CICAD (54.1%) and TFA-related CICAD (43.9%). Guide catheters and 6F catheters were used in approximately 80% of CICAD cases across both access site groups. There were 947 procedures during which a catheter greater than 6F was used, corresponding to 1.1% of the total number of catheterizations. As reported in Table 2, there were 5 cases of CICAD in these patients with greater than 6F catheters used—an incidence of 0.53% in this group with larger catheters than 0.16% in the overall cohort.

**Table 2:** Catheter-induced coronary artery dissection procedural details by access site.

	Total CICAD (N = 142)	Transradial access (n = 85)	Transfemoral access (n = 57)
Procedural characteristics			
STEMI at presentation	35 (24.6%)	23 (27.1%)	9 (15.8%)
Coronary artery involved in CICAD			
Left coronary artery dissection	52 (36.6%)	32 (37.7%)	20 (35.1%)
Right coronary artery dissection	71 (50%)	46 (54.1%)	25 (43.9%)
Isolated aortic dissection	10 (7.0%)	6 (7.1%)	4 (7.1%)
Left internal thoracic artery dissection	6 (4.2%)	1 (1.2%)	5 (8.8%)
Right internal thoracic artery dissection	3 (2.1%)	0 (0.0%)	3 (5.3%)
Catheter type			
Diagnostic	31 (21.8%)	15 (17.6%)	16 (28.1%)
Guide	110 (77.5%)	69 (81.2%)	41 (71.9%)
Unknown	1 (0.7%)	1 (1.2%)	0 (0.0%)
Catheter design			
Amplatz left	23 (16.2%)	11 (12.9%)	12 (21.1%)
Extra back-up	35 (24.6%)	22 (25.9%)	13 (22.8%)
Hockey stick	3 (2.1%)	0 (0.0%)	3 (5.3%)
Judkins left	15 (10.6%)	11 (12.9%)	4 (7.0%)
Judkins right	44 (31.0%)	27 (31.8%)	17 (29.8%)
Multiaortic curves	3 (2.1%)	3 (3.5%)	0 (0.0%)
Multipurpose	2 (1.4%)	1 (1.2%)	1 (1.8%)
Radial curve	1 (0.7%)	1 (1.2%)	0 (0.0%)
Special curve	7 (4.9%)	7 (8.2%)	0 (0.0%)
Internal mammary	7 (4.9%)	0 (0.0%)	7 (12.3%)
Ikari left	1 (0.7%)	1 (1.2%)	0 (0.0%)
Unknown	1 (0.7%)	1 (1.2%)	0 (0.0%)
Catheter size			
5F	13 (9.2%)	11 (12.9%)	2 (3.5%)
6F	123 (86.6%)	72 (84.7%)	51 (89.5%)
>6F	5 (3.5%)	1 (1.2%)	4 (7.0%)
Unknown	1 (0.7%)	1 (1.2%)	0 (0.0%)

Values are n (%).

CICAD, catheter-induced coronary artery dissection; STEMI, ST-elevation myocardial infarction.

In terms of absolute numbers, the extra back-up (15.0% usage in the overall cohort) and Judkins right (39.9% usage in overall cohort) were the 2 most common catheter designs associated with CICAD and comprised more than 50% of CICAD cases, followed by the Amplatz left (4.7% usage in overall cohort) and Judkins left (38.1% usage in overall cohort) (Table 2). The rate of CICAD per 10,000 uses of the most commonly utilized catheters was the greatest with the Amplatz left (126.1 cases of CICAD per 10,000 uses) followed by the extra back-up (21.0 cases of CICAD per 10,000 uses), Judkins left (9.9 cases of CICAD per 10,000 uses), and Judkins right (3.6 cases of CICAD per 10,000 uses).

In a sensitivity analysis, the association between access site and CICAD was evaluated from 2007-2011 (ie, TFA-predominant era) and 2012-2021 (ie, TRA-predominant era) (Central Illustration, Supplemental Table 2). In the TFA-predominant era, there were a total of 59 cases of CICAD, and TRA was associated with an increased risk of dissection (0.48% TRA vs 0.11% TFA; RR, 3.42; 95% CI, 2.05-5.69; *P* < .01). In the TRA-predominant era, there were 83 cases of CICAD, with 0.13% in the TRA group and 0.16% in the TFA group, and the association between access site and CICAD was no longer present (*P* = .40).

**Central Illustration:**
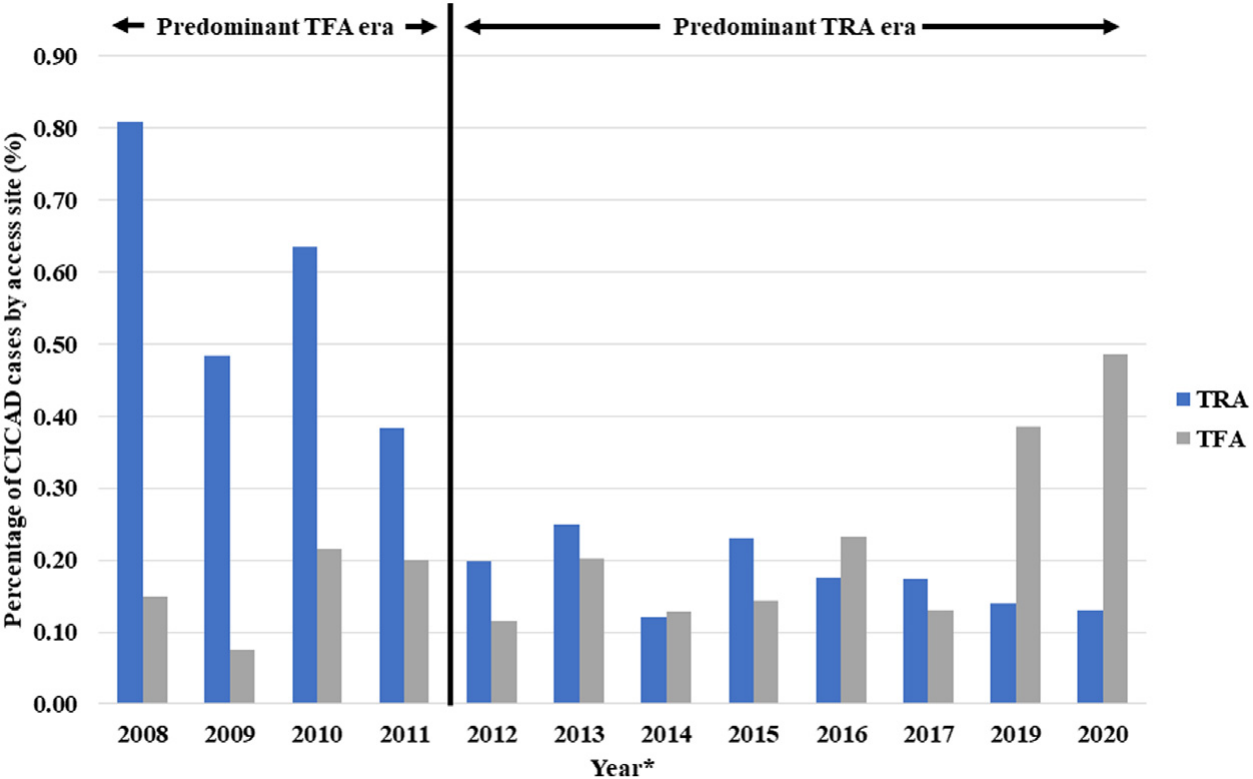
Percentage of catheter-induced coronary artery dissection cases over study period stratified by access site. In the TFA-predominant era, TRA was associated with an increased risk of dissection (0.48% TRA vs 0.11% TFA; RR 3.42, 95% CI 2.05-5.69, *P* < .01). In the TRA-predominant era, the association between access site and CICAD was no longer present (*P* = .40). ∗Only years in which there were more than 5 CICAD cases have been included in the figure CICAD, catheter-induced coronary artery dissection; TFA, transfemoral access; TRA, transradial access.

The management of CICAD involved bailout stenting in 99 (69.7%) cases and emergency coronary artery bypass graft (CABG) surgery in 18 (12.7%) cases (Table 3). There were 21 patients (14.8%) in whom the CICAD was felt to be self-limited and improved during intraprocedural observation—notably, the majority of these were isolated aortic injury (n = 12). Two patients required emergent mechanical circulatory support with veno-arterial extracorporeal membrane oxygenation prior to revascularization. Two patients (1.4%) experienced immediate death at the time of CICAD with inability to achieve revascularization. Of the 18 patients who were referred to CABG, 3 (17%) died during the surgery. In total, 12 patients (8.5%) with CICAD died in the hospital (Supplemental Table 3). Of these 12 patients who died in the hospital, 7 (58%) had retrograde extension of the CICAD into the aorta noted during the index procedure.

**Table 3:** Outcomes of patients with CICAD.

	Total CICAD (N = 142)
Management of CICAD	
Medical therapy	21 (14.8%)
Bailout stenting	99 (69.7%)
Coronary artery bypass graft surgery	18 (12.7%)
Extracorporeal membrane oxygenation prior to revascularization	2 (1.4%)
None–death prior to revascularization established	2 (1.4%)
Clinical outcomes	
Death during index procedure	2 (1.4%)
Death during coronary artery bypass graft surgery	3 (2.1%)
Death in-hospital	12 (8.5%)

Values are n (%).

CICAD, catheter-induced coronary artery dissection.

## Discussion

We report a series of 142 CICADs at our institution over a 15-year period. To the best of our knowledge, this represents the largest reported series of CICAD in the literature to date and the first to evaluate the incidence by vascular access site. CICAD remains a very rare complication of coronary angiography, with an incidence of 16 cases per 10,000 procedures in our series, similar to the incident rates previously reported in the literature.^9^, ^10^, ^11^, ^12^, ^13^

The TRA approach has a significant learning curve due to various factors including catheter manipulation, which is impeded by radial vasospasm, tortuous subclavian anatomy, and the use of catheters designed for TFA.^7^ Anecdotally, it has been felt that these factors increased the probability of suboptimal catheter engagement in noncoaxial or deep-seated positions, predisposing to CICAD. Similarly, torque introduced by catheter manipulation can transfer significant potential energy to the interface between the catheter and coronary wall. Despite these theoretical possibilities, we did not demonstrate an association between access site and CICAD over a 15-year period. However, when stratified by “predominant femoral access” between the years 2007-2011, we found that the risk of CICAD with TRA was nearly 3.5 times greater than with femoral access; however, this association was no longer present once our center had transitioned to "predominant radial access" (ie, 2012 and onwards). Similar to the initial concerns about increased procedural failure, contrast use, and radiation exposure, which have been refuted by studies showing that these parameters improve with operator experience,^7^^,^^8^ perhaps the risk of CICAD with radial access is also reduced as we progress along the learning curve of this approach. Of note, female sex was associated with an increased risk of CICAD in patients who underwent TRA. It has been hypothesized that hormonal imbalances may directly impact the coronary endothelium due to the presence of estrogen and progesterone receptors. This may result in decreased collagen production and ultimately weaken the arterial wall, culminating in increased susceptibility to dissection.^13^^,^^15^^,^^16^ Although this may increase the risk of dissection among females in general, we suspect that the reason this association was observed only in the TRA group is likely spurious.

The most common catheter designs associated with CICAD were the extra back-up and Judkins right. The Amplatz left catheter has been postulated to cause a disproportionate number of iatrogenic dissections.^17^ Our series corroborates this finding, with the Amplatz left design implicated in 16.2% of CICAD cases, although it was used in only 4.7% of all procedures. The special curve design was the CICAD culprit in 4.9% of cases, although it was used only in 0.3% of procedures (206 cases of CICAD per 10,000 uses of the catheter), suggesting it is a particularly risky catheter. Although the extra back-up was responsible for nearly a quarter of all CICAD, this likely represents its widespread use at our institution (15.0% of procedures). Similarly, while the Judkins catheters are responsible for a combined 42% of CICAD, they are used in nearly 80% of cases at our institution, suggesting they have a lower risk of CICAD. However, the Judkins catheters have both diagnostic and guide designs, which we were unable to differentiate in the “overall” group data, and the lower frequency of CICAD might simply be reflective of a lower risk with diagnostic catheters. Similarly, aggressive catheters such as the Amplatz left are used more frequently in complex diseases where more support is required, which may also increase the risk of CICAD.

Catheter-induced coronary artery dissection is a feared complication because of the perceived high morbidity and mortality.^11^ Conservative management, bailout stenting, and emergency CABG have all been described as CICAD treatment strategies.^9^, ^10^, ^11^, ^12^^,^^18^, ^19^, ^20^ In our series, the treatment strategy of choice was bailout stenting (69.7%) followed by conservative management (14.8%). The use of emergency CABG was less common (12.7%). CICAD was associated with 8.5% in-hospital mortality, which is slightly greater than previously reported.^11^^,^^13^ The reason for the discrepancy is unclear and may reflect differences in practice or reporting patterns at our institutions. In addition, of the 12 patients who died in the hospital, 7 (58%) had retrograde extension of the CICAD into the aorta noted during their procedure. Clinicians should maintain a high index of suspicion of aortic involvement with CICAD and have a low threshold to pursue imaging of the ascending aorta as this may be a potentially modifiable target for intervention following CICAD. Based on our data, the mortality of CICAD is substantial, and every step should be taken to mitigate the risk of this feared complication.

Our study is not without limitation. First, we relied on catheterization reports to identify cases of reported CICAD, which are likely underreported, and therefore, our work likely represents an underestimate of the incidence of this complication. Second, angiographic images were only available from 2014, thereby limiting our ability to grade the CICAD events according to the NHLBI system to ∼40% of CICAD cases.^21^ Similarly, we were unable to provide insight into the factors described by other authors that may contribute to the risk of dissection, including ascending aorta configuration, aberrant anatomy of the coronary ostium, presence of proximal atherosclerosis or plaque rupture or noncoaxial catheter engagement.^19^^,^^22^^,^^23^ Importantly, there were few universal catheters that permit both left and right coronary artery angiography used during the study period and therefore, this study does not exclude the possibility of higher CICAD with TRA with these catheters. Finally, this retrospective, observational study is hypothesis generating, and conclusions are limited by unforeseen bias and variables left unaccounted for.

## Conclusions

CICAD is a rare complication of coronary angiography and PCI. Over a 15-year period, we did not demonstrate an association between access site and the incidence of CICAD. There is considerable mortality associated with CICAD, and bailout stenting is the most common treatment, when possible. All operators must remain vigilant and mitigate the risk of CICAD whenever possible.
