# Supplementary material for: Association Between the Access Site for Coronary Angiography and Catheter-induced Coronary Artery Dissection
Source: J Soc Cardiovasc Angiogr Interv. 2023 Mar 4;2(3):100606. doi: 10.1016/j.jscai.2023.100606 (PMC11307617; doi:10.1016/j.jscai.2023.100606)
Supplement: Supplemental Appendix [file mmc1.docx]

**Supplemental Appendix**

**Supplemental Table 1:** National Heart, Lung and Blood Institute (NHLBI) classification

| **NHLBI class** | **Number of events** |
| --- | --- |
| Type A dissections (radiolucent areas within the coronary lumen during contrast injection, with minimal or no persistence of contrast) | 7 |
| Type B dissections (parallel tracts or double lumen separated by a radiolucent area during contrast injection, with minimal or no persistence) | 21 |
| Type C dissections (contrast outside the coronary lumen with persistence of contrast in the area after clearance of contrast from the coronary lumen) | 5 |
| Type D dissections (spiral luminal filling defects, frequently with extensive contrast staining of the vessel) | 5 |
| Type E dissections (new, persistent filling defects) | 1 |
| Type F dissections (lead to total occlusion of the coronary artery, without anterograde flow) | 15 |

**Supplemental Table 2** – Proportion of access site by transradial and transfemoral approaches stratified by year of the procedure*

| **Year** | **% TRA** | **% TFA** |
| --- | --- | --- |
| 2008 | 10% | 87% |
| 2009 | 13% | 84% |
| 2010 | 22% | 74% |
| 2011 | 46% | 51% |
| 2012 | 52% | 43% |
| 2013 | 53% | 41% |
| 2014 | 56% | 38% |
| 2015 | 62% | 33% |
| 2016 | 68% | 26% |
| 2017 | 71% | 23% |
| 2018 | 72% | 22% |
| 2019 | 81% | 15% |
| 2020 | 81% | 14% |

* - total per year may not equal 100% as procedures with multiple access sites were not included

**Supplemental Table 3** – Procedural characteristics of catheter-induced coronary artery dissection cases that resulted in in-hospital death

| **Year** | **Access site** | **Type of procedure** | **Catheter Design** | **Catheter Size** | **Artery involved** |
| --- | --- | --- | --- | --- | --- |
| 2008 | TRA | PCI | Multi-Aortic Curve | 6Fr | RCA |
| 2009 | TFA | PCI | Extra Back Up | 6Fr | LCA |
| 2009 | TRA | PCI | Amplatz Left | 6Fr | RCA |
| 2010 | TRA | PCI | Special Curve | 6Fr | RCA |
| 2011 | TRA | PCI | Extra Back Up | 6Fr | LCA |
| 2011 | TRA | PCI | Judkin’s Left | 6Fr | LCA |
| 2012 | TRA | PCI | Amplatz Left | 6Fr | RCA |
| 2015 | TRA | PCI | Judkin’s Right | 5Fr | RCA |
| 2017 | TFA | PCI | Amplatz Left | 6Fr | LCA |
| 2019 | TRA | PCI | Extra-Back Up | 6Fr | LCA |
| 2019 | TFA | PCI | Judkin’s Left | 6Fr | LCA |
| 2020 | TRA | PCI | Amplatz Left | 6Fr | RCA |

LCA = left coronary artery; PCI = percutaneous coronary intervention; RCA = right coronary artery; TFA = transfemoral access; TRA = transradial access
